# *AGTRAP* Is a Prognostic Biomarker Correlated With Immune Infiltration in Hepatocellular Carcinoma

**DOI:** 10.3389/fonc.2021.713017

**Published:** 2021-09-14

**Authors:** Shanshan Liu, Wei Zhao, Xuemei Li, La Zhang, Yu Gao, Qiling Peng, Chengyou Du, Ning Jiang

**Affiliations:** ^1^Department of Hepatobiliary Surgery, The First Affiliated Hospital of Chongqing Medical University, Chongqing, China; ^2^School of Basic Medical Science, Chongqing Medical University, Chongqing, China; ^3^Department of Pathology, Chongqing Medical University, Chongqing, China

**Keywords:** hepatocellular carcinoma, *AGTRAP*, prognosis, immune infiltration, biomarker

## Abstract

**Background:**

Recently, it has been reported that angiotensin II receptor-associated protein (*AGTRAP*) plays a substantial role in tumor progression. Nevertheless, the possible role of *AGTRAP* in hepatocellular carcinoma (HCC) remains unrecognized.

**Methods:**

The metabolic gene rapid visualizer, Cancer Cell Line Encyclopedia, Human Protein Atlas, and Hepatocellular Carcinoma Database were used to analyze the expression of *AGTRAP* in HCC tissues and normal liver tissues or adjacent tissues. Kaplan-Meier plotter and UALCAN analysis were used to assess the prognostic and diagnostic value of *AGTRAP.* LinkedOmics and cBioPortal were used to explore the genes co-expressed with *AGTRAP* in HCC. To further understand the potential mechanism of *AGTRAP* in HCC, Gene Ontology and Kyoto Encyclopedia of Genes and Genomes enrichment pathway analyses were performed using *R* software, the protein-protein interaction (PPI) network was established using the STRING database, and the immune infiltration and T-cell exhaustion related to *AGTRAP* were explored *via* Timer and GEPIA. In addition, immunohistochemistry was used to detect the expression of AGTRAP protein in HCC tissues and paired adjacent tissues from clinical specimens.

**Results:**

This study found that the mRNA and protein levels of AGTRAP in HCC tissues were higher than those in normal liver tissues and adjacent tissues, and higher mRNA levels of *AGTRAP* were associated with higher histological grade and a poor overall survival in HCC patients. The area under the receiver operating characteristic curve (AUC) of *AGTRAP* was 0.856, suggesting that it could be a diagnostic marker for HCC. Moreover, the alteration rate of *AGTRAP* in HCC was 8%, and *AGTRAP* was involved in HCC probably through the NF-κB and MAPK signaling pathways. Furthermore, *AGTRAP* was positively correlated with the infiltration of CD8^+^ T cells, CD4^+^ T cells, B cells, macrophages, dendritic cells, and neutrophils, and the levels of *AGTRAP* were significantly correlated with T-cell exhaustion biomarkers. The immunohistochemistry results confirmed that the protein levels of AGTRAP were consistently higher in HCC tissues than in paired adjacent tissues.

**Conclusion:**

The clinical value of *AGTRAP* and its correlation with immune infiltration in HCC was effectively identified in clinical data from multiple recognized databases. These findings indicate that *AGTRAP* could serve as a potential biomarker in the treatment of HCC, thereby informing its prognosis, diagnosis, and even immunotherapy.

## Introduction

Hepatocellular carcinoma (HCC) is the fourth most common malignant tumor worldwide (1) with low overall survival (OS) of patients and poor prognosis (2). The majority of patients are diagnosed with HCC at an advanced stage, usually accompanied by intra- and extra-hepatic invasion and metastasis, with no specific symptoms at the early stage (3). Although an increasing number of adjuvant therapeutic methods have been found to improve the treatment efficacy of HCC, the recurrence and metastasis of HCC remain difficult obstacles. Therefore, there is an urgent need to identify novel specific biomarkers to improve the clinical diagnosis and prognosis in HCC patients.

Angiotensin II receptor-associated protein (*AGTRAP*) is localized in the perinuclear vesicle structure and the plasma membrane (4). *AGTRAP*, which inhibits the activation of angiotensin II, has been broadly studied in cardiovascular diseases and metabolic disorders (5, 6). Recently, Sanz-Pamplona et al. (7) found that *AGTRAP* is overexpressed in colon cancer and positively correlates with a poor prognosis. Figueiredo et al. (8) reported that A*GTRAP-BRAF* gene fusion was detected in gastric cancer, indicating a possible role of *AGTRAP* in tumor progression. However, little is known about the function of *AGTRAP* in HCC and the oncogenic mechanisms involved.

Bioinformatics is a combination of life science and computer science that aims to identify the sequence, structure, and function of biological macromolecules and their interrelationships. In the last several years, bioinformatics has proved to be a valuable tool in screening tumor biomarkers (9, 10). In this study, we used bioinformatics to explore the expression of *AGTRAP* in HCC tissues and normal tissues or adjacent tissues, the value of *AGTRAP* in HCC patient prognosis and diagnosis, and its correlation with clinicopathological parameters, and performed enrichment analyses of *AGTRAP* alterations and its co-expressed genes using Gene Ontology (GO) and Kyoto Encyclopedia of Genes and Genomes (KEGG). Finally, immunohistochemistry (IHC) was used to verify the levels of *AGTRAP* in HCC tissues and paired adjacent tissues. Our study aimed at defining the potential role of *AGTRAP* in the occurrence and progression of HCC and whether it could be used as a diagnostic and prognostic biomarker.

## Materials And Methods

### TCGA Database

The gene expression RNA-Seq (HTSeq-FPKM), clinical data, and survival data were downloaded from the The Cancer Genome Atlas (TCGA) database from UCSC Xena (https://xenabro.wser.net/datapages/), which are processed uniformly by the TOIL process, free of computational batch effects (11).

### Metabolic Gene Rapid Visualizer and Cancer Cell Line Encyclopedia

The Metabolic Gene Rapid Visualizer (MERAV, http://merav.wi.mit.edu) database was used to investigate the expression of *AGTRAP* (12). The gene expression data are normalized across these arrays to provide a means of consistent comparison. The Cancer Cell Line Encyclopedia (CCLE, https://portals.broadinstitute.org/ccle) integrates genetic information, including chromosome copy number, gene expression, and DNA mutations. CCLE can be applied to the evaluation of cellular targets, small molecules, genetic variations, and therapeutic approaches, allowing the recognition of new biomarker drivers of tumor dependence. The CCLE database provides a resource for cancer studies using models of cancer cell lines *in vitro* (13).

### Hepatocellular Carcinoma Database and the Human Protein Atlas

In addition, the expression of *AGTRAP* in different liver tissues was studied using the Hepatocellular Carcinoma Database (HCCDB, http://lifeome.net/database/hccdb) (14). Simultaneously, co-expression networks of adjacent tissues and HCC tissues were assessed through HCCDB. Datas were normalized by log2 FC, and P-values were adjusted with Benjamini & Hochberg correction.

The protein expression of AGTRAP in both LIHC and normal tissues was obtained from the Human Protein Atlas database (HPA, version 20.1) (https://www.proteinatlas.org/), which is a program with the aim to map all the human proteins in cells, tissues. Next, the HPA was used to analyze the expression of AGTRAP in human tissues and cells (15).

### Kaplan-Meier Plotter and UALCAN

The Kaplan-Meier plotter (http://kmplot.com/analysis/) (16) was used to analyze the prognostic value of *AGTRAP* in HCC samples based on TCGA (https://www.cancer.gov/) datasets.

UALCAN (ualcan.path.uab.edu) (17) is an open database for the analysis of gene expression data (TPM) from TCGA, which has been normalized. We used UALCAN to analyze the difference in *AGTRAP* expression in normal and HCC samples, the relationships between *AGTRAP* expression and the clinicopathological parameters of each sample (tumor grade, tumor stage, TP53 mutant, weight, age, sex, nodal metastasis), and its potential prognostic value.

### LinkedOmics and cBioPortal

LinkedOmics (https://linkedomics.org/) is an online analysis tool that can be used to compare multi-omic cancer datasets from the Clinical Proteomic Tumor Analysis Consortium (CPTAC) and TCGA with an array of tumor types (32 cancers and 11,158 patients) and proteomics data (18). LinkedOmics involves three important analysis modules (LinkCompare, LinkFinder, and LinkInterpreter) and plots data as heat maps, scatter plots, and volcano plots. We selected RNAseq data from the TCGA-LIHC dataset for analysis, which are RSEM format data that has been converted by log2, using the Pearson correlation test, and they corrected for false positives (False Discovery Rate, FDR) using the Benjamini-Hochberg (BH) correction.

The cBio Cancer Genomics Portal (cBioPortal, version 3.7.1, http://cbioportal.org) was principally applied to explore multidimensional cancer genomics datasets with resources from 20 cancer studies including more than 5,000 tumor samples (19). We selected the log RNA Seq V2 RSEM data from TCGA-LIHC on this website for mutation analysis and co-expression gene analysis, which has been normalized.

### STRING

The Search Tool for the Retrieval of Interacting Genes (STRING, version 11.5, http://string-db.org) provides references for studying the mechanism of disease occurrence or progression and provides a basis for exploring the functional interactions between proteins (20). STRING was used to demonstrate the functional networks of AGTRAP in HCC.

### TIMER and GEPIA

The Tumor Immune Estimation Resource (TIMER, version 1.0, https://cistrome.shinyapps.io/timer/) was applied to analyze the association between *AGTRAP* gene expression and immune infiltration in HCC, as well as T-cell exhaustion (21). The relevant immune cells include B Cells, CD8^+^ T Cells, CD4^+^ T Cells, macrophages, neutrophils, and dendritic cells. The scatterplots will be generated and displayed after inputs are submitted successfully, showing the purity-corrected partial Spearman’s rho value and statistical significance.

The Gene Expression Profiling Interactive Analysis (GEPIA, version 1.0, http://gepia.cancer-pku.cn/) is a newly developed interactive web server for analyzing the RNA sequencing expression data of 9,736 tumors and 8,587 normal samples from the TCGA and the GTEx projects, using a standard processing pipeline. The RNA-Seq datasets GEPIA used is based on the UCSC Xena project (http://xena.ucsc.edu), which are computed by a standard pipeline. The database provides a customizable and interactive function, including differential expression analysis, correlation analysis, profiling plots, gene analysis, and survival analysis. The associations between *AGTRAP* levels and T-cell exhaustion biomarkers (PD-1 (PDCD1), GZMB, LAG-3, CTLA-4, and HAVCR2 (TIM-3)) were explored using the GEPIA database (22). The data uses Log2 (TPM+1) for normalization. We performed a survival analysis of the TCGA-LIHC dataset with a median cutoff, Kaplan–Meier (KM) plots are presented with the hazard ratio (HR), the 95% confidence interval (CI), the log-rank *p*-value (*p*). Pearson correlation was chosen for gene correlation analysis.

### Immunohistochemistry Analysis

The study was approved by the Ethics Committee of the First Affiliated Hospital of Chongqing Medical University. All participants provided informed consent in writing. The pathological sections of 10 HCC patients were re-analyzed by professional pathologists *via* IHC staining of AGTRAP in paired tumors and adjacent tissues using the streptavidin-peroxidase (SP) method. Paraffin-embedded tumor tissues were collected and sliced into 3 mm sections. The slices were baked at 60°C for 2 h to be fully dewaxed and then rehydrated through a graded alcohol series and washed with phosphate buffered saline. Hydrogen peroxide (3%) was used to block endogenous peroxidase activity for 10 min at 25°C. Antigen retrieval was performed by microwaving in 10 mM citrate buffer (pH = 6.0) for 15 min. The slides were then blocked and sealed with 5% normal goat serum for 30 min at room temperature. The rest of the procedures are in accordance with the kit instructions (SP-9000, OriGene, China). Fifty microliters of anti-*AGTRAP* antibody (1:100, Sangon Biotech, China) was added to these tissue sections, which were then incubated at 4°C overnight. The sections were then incubated with the corresponding secondary antibody, OriGene, for 30 min at room temperature. Finally, the IHC score for each sample was calculated as the intensity of immunostaining (0, colorless; 1, light-yellow; 2, brownish-yellow; 3, dark brown) multiplied by the percentage of positive cells (0: 0%, 1: 1%–10%, 2: 11%–50%, 3: 51%–75%, and 4: 76%–100%).

### Western Blotting

Radioimmunoprecipitation assay buffer (RIPA) buffer purchased from Beyotime Biotechnology (China) was used to extract total protein from tissues, and the supernatant was collected after centrifugation (12,000×g, 15 min, 4°C). BCA Protein Assay Kit (Biyuntian, China) was used to quantify the protein concentration. Equal protein quantities were separated by 12.5% SDS-PAGE gel and then transferred to PVDF membranes (0.22 um, PVDF membrane, GE healthcare life science, Germany). Membranes were blocked with 5% skimmed milk in Tris-buffered saline (TBS) for 1 h and was incubated with primary antibodies against AGTRAP (1:2000, ABclonal, China) and β-actin (1:2000, ABclonal, China) at 4°C overnight. The membrane was then incubated with secondary antibodies (1:2,000, Proteintech, China) for 1 h the next day. Finally, the signals were developed by an ECL detection system (Enhanced chemiluminescence system kit). Subsequently, gray scale quantifications of bands were performed using Image J software (National Institutes of Health, USA).

### Statistical Analysis

All statistical analyses were performed using the SPSS software (version 26.0, IBM, USA) and the GraphPad Prism software (version 7.0, USA, www.graphpad.com) was used for graphing the heatmap and analyze the different grayscale values between adjacent tissues and tumor tissues of western blot bands. The Receiver operating characteristic curve (ROC) was analyzed using the “pROC” package (version 1.17.0.1) and “ggplot2” (version 3.3.3) in R Studio (version 3.6.3). The GO and KEGG enrichment analyses were visualized using the R packages “ggplot2” (version 3.3.3) and “clusterProfiler” (version 3.15.3), the threshold for statistically significant differences was set at *p adjust ≤0.05.* A chi-square test was used to assess the correlation between *AGTRAP* expression and clinicopathological parameters. Logistic regression and Spearman correlation analyses were used to analyze the correlation between clinical parameters and *AGTRAP* expression. Univariate and multivariate Cox regression analyses were performed to determine the association between *AGTRAP* expression and OS. Differences were considered statistically significant at *p < 0.05*.  *F*alse discover rate (FDR) < 0.05 was regarded statistically credible.

## Results

### The mRNA and Protein Expression of *AGTRAP* in Different Databases

**Figure 1:**
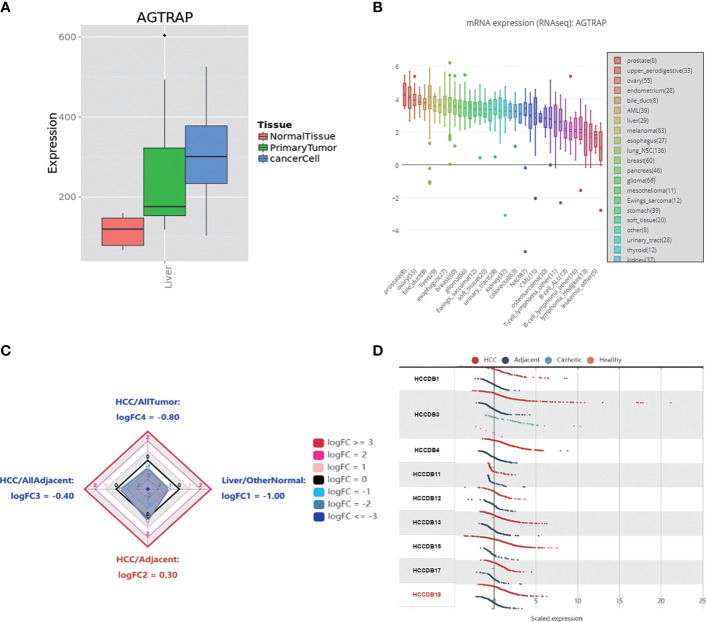
The mRNA expression of *AGTRAP* in different datasets. **(A)** The mRNA expression level of *AGTRAP* was significantly higher in HCC cells and Primary liver tissues than that in normal tissues in the MERAV. **(B)** Expression of *AGTRAP* in HCC cell lines using the CCLE database. **(C)** Radar map of *AGTRAP* overall expression among different types of tissues. **(D)** The expression of *AGTRAP* in different HCC datasets.

First, we explored the different expression levels of *AGTRAP* mRNA in HCC cells and HCC tissues as well as normal tissues in the MERAV (**Figure 1A**). *AGTRAP* expression was significantly higher in HCC cells than in normal tissue samples. The CCLE database was then used to visualize the gene expression data. As assessed through an array of cancer cell lines, *AGTRAP* was highly expressed in HCC cells (**Figure 1B**). Next, we reviewed the HCCDB to identify whether there were significant differences in *AGTRAP* mRNA expression levels between HCC datasets. As shown in **Figure 1C**, the *AGTRAP* level in HCC tissues was higher than in adjacent tissues (HCC/adjacent: logFC = 0.30). Subsequently, we compared *AGTRAP* levels in 12 different HCC datasets of which 9 supported our results (**Figure 1D**).

**Figure 2:**
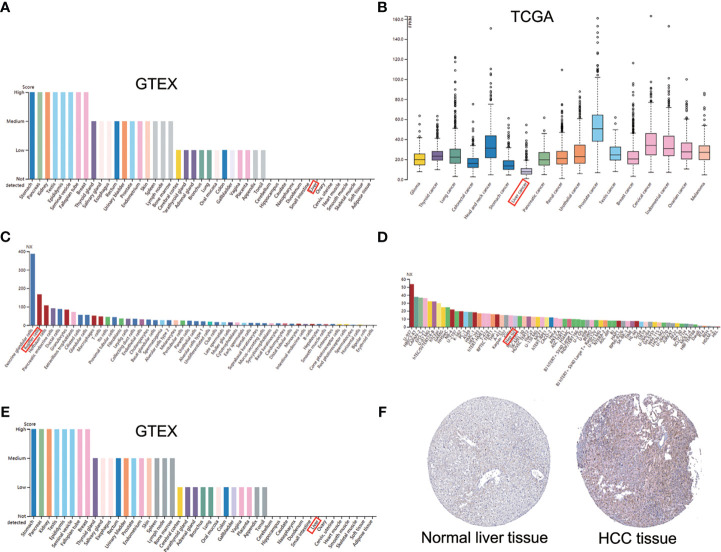
The mRNA and protein expression of AGTRAP in human normal tissues, cancer tissues and cell lines in HPA database. **(A)** histogram of *AGTRAP* mRNA expression in kinds of normal tissues from the GTEx project. **(B)** *AGTRAP* mRNA expression in different tumor tissues. **(C)** mRNA expression of *AGTRAP* in HCC cell lines (HEP G2). **(D)** The expression of *AGTRAP* in single cells. **(E)** Protein expression of AGTRAP in normal tissues from different organs. **(F)** IHC images with the AGTRAP antibody: HPA044120, HCC tissues had higher staining than normal liver tissues.

*AGTRAP* expression in normal liver tissues was lower compared to majority of human tissues in HPA (**Figure 2A**). Additionally, the level of *AGTRAP* in HCC tissues was the lowest among the cancer samples (**Figure 2B**). However, *AGTRAP* levels were higher in HCC cell lines (Hep G2) than in most cancer cell lines (**Figure 2C**). Intriguingly, the expression of *AGTRAP* in liver cells was mainly concentrated in Kupffer cells, and its expression was also high in T cells (**Figure 2D**). Although the expression of *AGTRAP* was relatively low in normal human liver tissues (**Figure 2E**), the HPA database showed that AGTRAP protein was moderately expressed in HCC tissues by IHC, mainly localized in cell plasma (**Figure 2F**).

### High *AGTRAP* Expression Is Correlated With Poor OS in HCC Patients and May Serve as a Diagnostic Biomarker Based on TCGA Datasets

**Figure 3:**
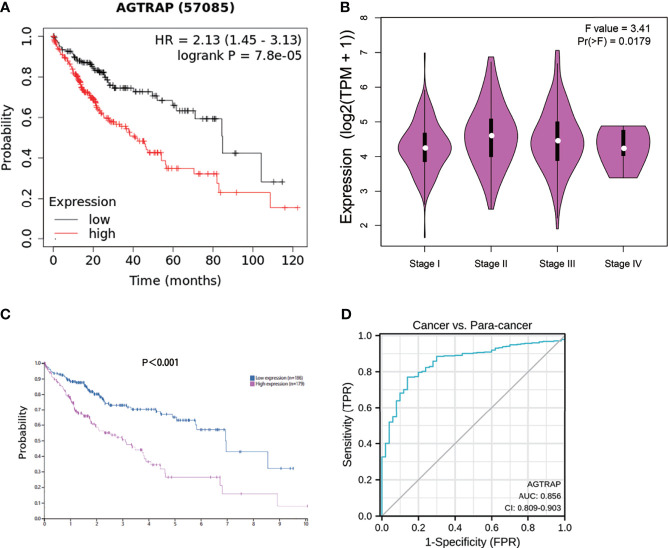
The correlation between the expression of *AGTRAP* mRNA and protein with overall survival and tumor stage in HCC. **(A)** Higher *AGTRAP* mRNA expression indicated a poor OS in HCC patients from the Kaplan-Meier plotter. **(B)** Expression violin plots of *AGTRAP* based on patient pathological stage [log2(TPM + 1)]. **(C)** Prognostic value of *AGTRAP* expression in HCC patients according to HPA. **(D)** ROC curves of *AGTRAP* in HCC.

To explore the association between *AGTRAP* level and OS in HCC patients, we used the Kaplan-Meier plotter tool and found a significant correlation between high expression of *AGTRAP* and poor prognosis (**Figure 3A**, hazard ratio (HR) = 2.13, 95% CI = 1.45–3.03, *p = 7.8e-5*). Next, we conducted a correlation analysis in the GEPIA database. The resultant violin plots showed that expression of *AGTRAP* was significantly correlated with the pathological stage of patients (**Figure 3B**). These findings compare well to HCC patient OS data from the HPA indicating that the high expression of *AGTRAP* was linked to poor prognosis (**Figure 3C**). Based on the TCGA dataset, we constructed an ROC using the pROC package (23) and found an AUC of 0.856 (**Figure 3D**).

### *AGTRAP* mRNA Expression Is Associated With Clinicopathological Characteristics in HCC Patients Based on TCGA Datasets

**Figure 4:**
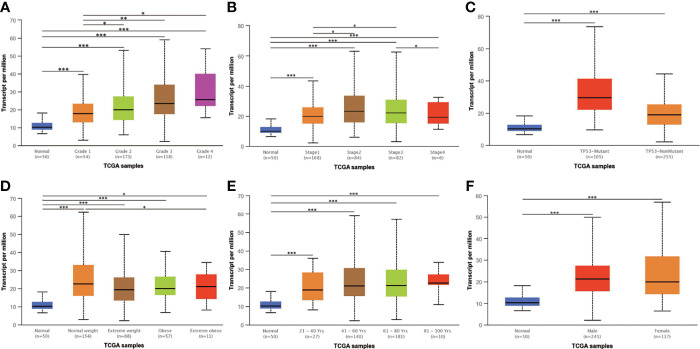
The expression of *AGTRAP* mRNA in the UALCAN database. *AGTRAP* expression was positively correlated with **(A)** tumor grade, **(B)** tumor stage, **(C)** TP53 mutation status, and **(D)** patient weight. No significant differences were found in **(E)** age and **(F)** gender. * represents *p < 0.05*, ** represents *p < 0.01*, *** represents *p < 0.001*.

The UALCAN database was used to explore whether *AGTRAP* mRNA expression is associated with clinicopathological parameters. As illustrated in **Figure 4A**, a positive trend was found between expression of *AGTRAP* in HCC and the progression of tumor grade. The analysis of additional parameters found *AGTRAP* mRNA levels were positively correlated with tumor stage, patient weight, and TP53 mutation (**Figures 4B** **–D**). Furthermore, the expression of *AGTRAP* mRNA in cancer tissues was higher than that in normal tissues (**Supplementary Figure 1A**). There were no statistical differences between the expression of *AGTRAP* and different age periods, gender, and lymph node metastasis status (**Figures 4E, F** and **Supplementary Figure 1B**).

### The Correlation Between Clinical Features and *AGTRAP* Expression in HCC From TCGA Data

To further analyze the clinical relevance of *AGTRAP* expression in HCC, we used TCGA clinical parameter data related to *AGTRAP* expression and deleted cases with missing data. The data was divided into two patient groups based on the median *AGTRAP* expression level (high or low) and the differences in group clinical features were analyzed using a chi-square test (**Table 1**). We found that histological grade varied significantly between the low and high expression groups. Logistic regression analysis and Spearman correlation analysis were used to analyze the correlation between these clinical parameters and the expression level of *AGTRAP* (**Tables 2**, **3**) and indicated a strong correlation between OS time and *AGTRAP* level. Univariate Cox regression analyses showed that age, race, pathologic stage, histologic grade, and T stage were related to OS. Finally, significant factors were analyzed by multivariate Cox regression which indicated that *AGTRAP* was an independent risk factor affecting the OS of patients with HCC (**Tables 4**, **5**).

**Table 1:** Associations between *AGTRAP* mRNA expression and clinicopathological characteristics in HCC.

Characteristics	cases	Low expression of *AGTRAP* (%)	High expression of *AGTRAP* (%)	P value
		134 (50.2)	133 (49.8)	
Age				0.762
<=60	126	64 (24.0)	62 (23.2)	
>60	141	69 (25.8)	72 (27.0)	
Gender				0.965
Female	92	46 (17.2)	46 (17.2)	
Male	175	87 (32.6)	88 (33.0)	
Weight				0.126
<=70	141	64 (24.0)	77 (28.8)	
>70	126	69 (25.8)	57 (21.3)	
Race				0.942
Asian	120	61 (22.8)	59 (22.1)	
Black or African American	11	5 (1.9)	6 (2.2)	
White	128	64 (24.0)	64 (24.0)	
Pathologic stage				0.145
Stage I	250	181 (67.8)	69 (25.8)	
Stage II	60	22 (8.2)	38 (14.2)	
Stage III	53	28 (10.5)	25 (9.4)	
Stage IV	4	2 (0.7)	2 (0.7)	
T stage				0.146
T1	152	82 (30.7)	70 (26.2)	
T2	60	22 (8.2)	38 (14.2)	
T3	51	27 (10.1)	24 (9.0)	
T4	4	2 (0.7)	2 (0.7)	
N stage				0.996
N0	265	132 (49.4)	133 (49.8)	
N1	2	1 (0.4)	1 (0.4)	
M stage				0.994
M0	264	131 (49.1)	133 (49.8)	
M1	4	2 (0.7)	2 (0.7)	
Histologic grade				0.032^*^
G1	22	13 (24.0)	9 (3.4)	
G2	133	76 (25.8)	57 (21.3)	
G3	100	40 (13.6)	60 (22.5)	
G4	12	4 (1.1)	8 (3.0)	
AFP (ng/ml)				0.335
<=400	202	104 (41.1)	98 (36.7)	
>400	125	29 (10.7)	96 (36.0)	
Albumin (g/dl)				0.084
<3.5	60	24 (13)	36 (13.5)	
>=3.5	207	109 (39.3)	98 (36.7)	

^*^ means p<0.05.

**Table 2:** Logistic regression analysis of the risk factors of *AGTRAP* high expression in HCC.

Characteristics	OR	95% CI	P value
Age	1.015	0.99-1.04	0.185
Gender	0.815	0.43-1.54	0.530
Weight	0.988	0.97-1.00	0.151
Race			0.395
Asian			
Black or African American	0.604	0.29-1.25	0.174
White	0.808	0.21-3.14	0.758
T stage			0.165
T1			
T2	0.619	0.06-6.52	0.690
T3	1.336	0.12-14.55	0.812
T4	0.728	0.07-7.60	0.791
N stage	0.667	0.04-11.89	0.783
M stage	1.147	0.11-12.18	0.909
Histologic grade			0.044^*^
G1			
G2	0.296	0.06-1.56	0.151
G3	0.324	0.08-1.26	0.104
G4	0.729	0.19-2.73	0.639
AFP (ng/ml)	1.000	1.00-1.00	0.466
Albumin (g/dl)	1.001	0.99-1.01	0.795
OS time	0.999	1.00-1.00	0.001^**^

* means p<0.05, ** means p<0.01.

**Table 3:** Spearman analysis of correlation between *AGTRAP* and clinicopathological parameters.

Characteristics	*AGTRAP* expression level	
	Spearman Correlation	p value
Age	-0.017	0.781
Weight	-0.107	0.082
Albumin	-0.056	0.363
AFP	0.125	0.041^*^
OS time	-0.293	0.000^***^

* means p<0.05, *** means p<0.001.

**Table 4:** Univariate Cox Regression Analysis on Survival of 267 HCC patients in TCGA dataset.

Variables	Univariate analysis		
	HR	95% CI	P value
Age	1.023	1.00-1.04	0.018^*^
Gender	1.528	0.98-2.37	0.060
Weight	0.994	0.99-1.01	0.979
Race			0.002^**^
Asian			
Black or African American	0.451	0.27-0.75	0.002^**^
White	1.829	0.72-4.63	0.203
Pathologic stage			0.001^**^
Stage I			
Stage II	0.152	0.05-0.43	0.000^***^
Stage III	0.144	0.05-0.44	0.001^**^
Stage IV	0.267	0.09-0.78	0.016^*^
T stage			0.025^*^
T1			
T2	0.255	0.08-0.83	0.023^*^
T3	0.233	0.07-0.82	0.023^*^
T4	0.436	0.13-1.47	0.180
N stage	0.339	0.08-1.39	0.133
M stage	0.173	0.06-0.48	0.001^**^
Histologic grade			0.199
G1			
G2	0.246	0.06-0.48	0.039^*^
G3	0.503	0.20-1.29	0.152
G4	0.584	0.23-1.51	0.266
AFP (ng/ml)	1.000	1.00-1.00	0.212
Albumin (g/dl)	0.998	0.91-1.09	0.966
*AGTRAP*	1.450	1.13-1.87	0.004^**^

* means p<0.05, ** means p<0.01, *** means p<0.001.

**Table 5:** Multivariate Cox Regression Analysis on Survival of 267 HCC patients in TCGA dataset.

Variables	Multivariate analysis		
	HR	95% CI	P value
Age	1.022	1.00-1.04	0.029^*^
Race			0.007^**^
Asian			
Black or African American	0.559	0.33-0.96	0.035^*^
White	2.455	0.95-6.38	0.065
Pathologic stage			0.625
Stage I			
Stage II	0.583	0.14-2.51	0.469
Stage III	2.022	0.21-9.58	0.543
T stage			
T1			
T2	4.544	0.35-59.25	0.248
T4	4.024	0.46-35.26	0.209
M stage	0.094	0.01-0.64	0.016^*^
*AGTRAP*	1.448	1.10-1.90	0.007^**^

* means p<0.05, ** means p<0.01.

### GO and KEGG Analyses of *AGTRAP* and Its Co-Expressed Genes in HCC

**Figure 5:**
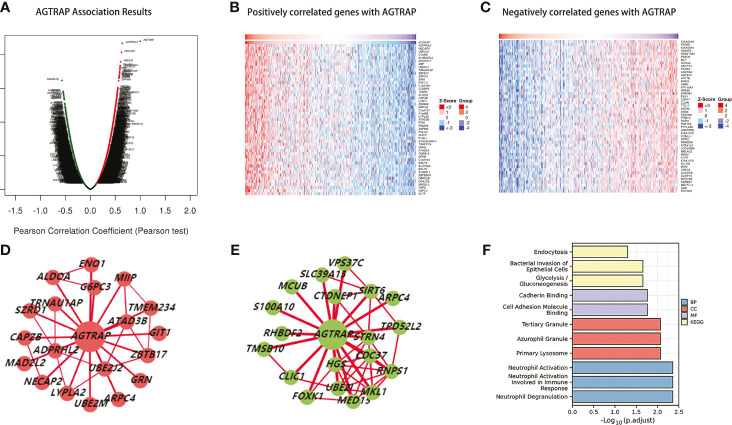
Genes in correlation with *AGTRAP* in HCC (LinkedOmics). **(A)** Genes correlated with *AGTRAP* showing in volcano plot; red (positive), green (negative). **(B, C)** Heat maps showing the top 50 genes positively and negatively correlated with *AGTRAP* in HCC. **(D)** The co-expression networks of *AGTRAP* in HCC tissues. **(E)** The co-expression networks of *AGTRAP* in adjacent liver tissues. **(F)** GO and KEGG enrichment analysis of genes co-expressed in HCC tissues and adjacent liver tissues.

To better understand the biological role of *AGTRAP*, the LinkedOmics database was used to identify co-expressed genes that are both positively and negatively correlated with *AGTRAP* (**Figure 5A**). Heat maps were used to visualize the 50 genes with the strongest correlation to *AGTRAP* (**Figures 5B, C**). Next, using the HCCDB, we analyzed the co-expression networks of *AGTRAP* and found significant differences between HCC tissues and adjacent liver tissues (**Figures 5D, E**). In addition, we performed GO and KEGG enrichment analyses of the co-expressed genes in HCC tissues and adjacent liver tissues (24). The main biological processes these genes were involved in were neutrophil degranulation, neutrophil activation involved in immune response, and neutrophil activation. These genes were located in the primary lysosome, azurophil granule, and tertiary granule. Additionally, they also play crucial roles in cadherin binding and cell adhesion molecule binding. Lastly, KEGG pathway analyses indicated that these genes were mainly enriched in glycolysis/gluconeogenesis, bacterial invasion of epithelial cells, and endocytosis (**Figure 5F**).

### GO and KEGG Analyses of *AGTRAP* and Its Neighboring Genes Associated With *AGTRAP* Alterations in HCC

**Figure 6:**
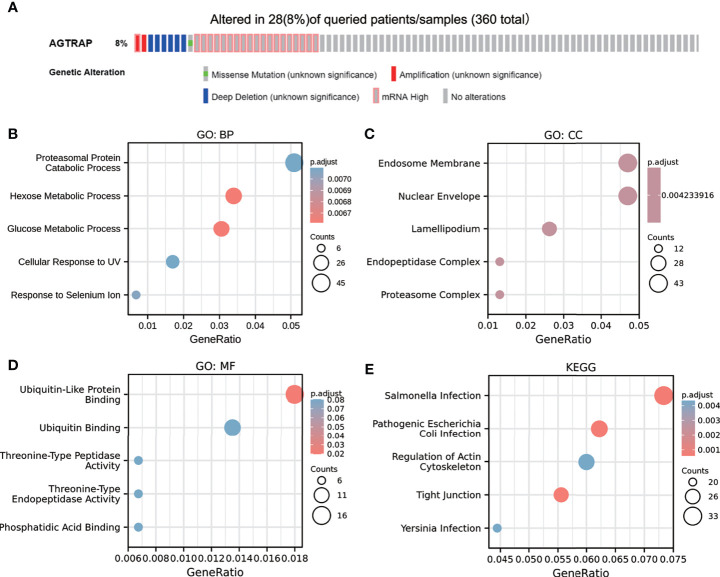
Genetic alterations of *AGTRAP* (cBioPortal) and the GO and KEGG analyses of the neighboring genes associated with *AGTRAP* alterations in HCC. **(A)** The mutation rate of *AGTRAP* was 8%. The significantly enriched GO annotations: **(B)** biological processes, **(C)** cellular components, **(D)** molecular functions, and **(E)** KEGG pathways of *AGTRAP* neighboring genes.

Considering that genomic alterations of *AGTRAP* are pathogenic, the CBioPortal database was used to investigate genetic alterations of *AGTRAP* based on TCGA sequencing data. *AGTRAP* was altered in 28 of the 360 (8%) patients. From the results there was 1 patient with high *AGTRAP* mRNA expression (0.29%), 1 patient with amplification (0.29%), 6 patients with deep deletion (1.67%), 1 patient with missense mutation (0.29%), 17 patients with high mRNA mutation (4.72%), and only one patient with two alterations (0.29%) (**Figure 6A**).

To further analyze the biological function of the *AGTRAP* gene and its neighboring genes, we performed GO and KEGG analyses on the genes that were positively correlated with *AGTRAP* gene alteration. The results showed that these genes were mainly enriched in Salmonella infection, pathogenic *Escherichia coli* infection, regulation of actin cytoskeleton, tight junction, and Yersinia validated infection (**Figures 6B** **–E**).

### Establishment of the PPI Network

**Figure 7:**
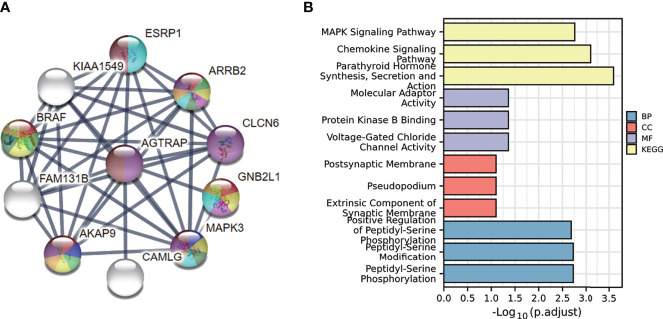
PPI of AGTRAP in HCC patients and the GO and KEGG analyses of these genes in HCC. **(A)** PPI of AGTRAP and other interacting proteins. **(B)** GO and KEGG enrichment analyses of genes interacted with AGTRAP in the String database.

To further understand the mechanisms of this gene, we used the STRING database to conduct a protein-protein interaction network analysis and found the strongest interactions with MAPK3, B-RAF, ARRB2, CLCN6, GNB2L1, and AKAP9 proteins (**Figure 7A**). We then performed enrichment analysis of the genes associated with these interacting proteins. The results showed that they were mainly enriched in parathyroid hormone synthesis, secretion and action, chemokine signaling pathway, and MAPK signaling pathway (**Figure 7B**).

### *AGTRAP* Overexpression Is Associated With Immune Infiltration and T-Cell Exhaustion

**Figure 8:**
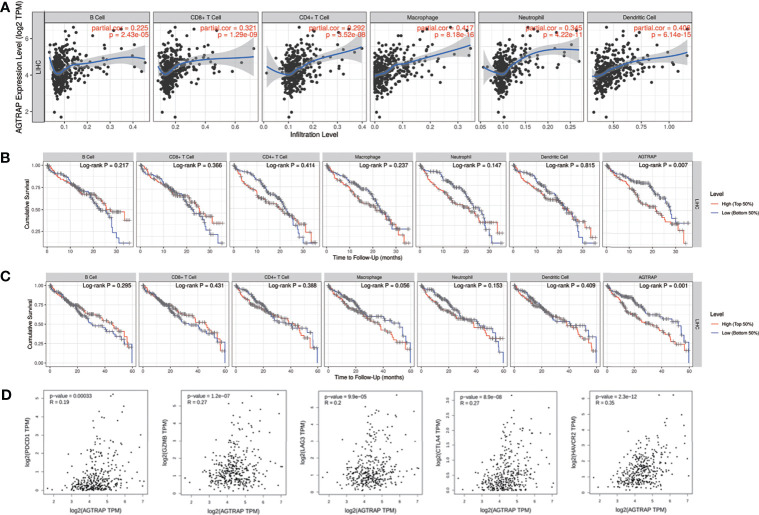
Association between *AGTRAP* and immune infiltration and T cell exhaustion in HCC. **(A)** *AGTRAP* is correlated with immune infiltrations in HCC. **(B, C)** Analysis of 3-year and 5-year survival of immune infiltration and *AGTRAP* expression in patients with HCC. **(D)** The correlation between *AGTRAP* expression and T cell exhaustion in HCC analyzed by the GEPIA database.

As shown in the HPA database, *AGTRAP* was highly expressed in Kupffer cells and T cells. We suggested that this was related to immune infiltration and T-cell exhaustion. The relationship between *AGTRAP* level and immune infiltration and T-cell exhaustion in HCC was explored using the TIMER online tool based on TCGA data. *AGTRAP* expression was positively correlated with the infiltration of B cells (correlation = 0.225, *p = 2.43e-05*), CD8^+^ T cells (correlation = 0.321, *p = 1.29e-09*), CD4^+^ T cells (correlation = 0.292, *p = 3.52e-08*), macrophages (correlation = 0.417, p = 8.18e-16), neutrophils (correlation = 0.345, *p = 4.22e-11*), and dendrites (correlation = 0.406, *p = 6.14e-15*) (**Figure 8A**). It also correlated with the 3-year (p = 0.007) and 5-year survival rates (*p = 0.001*) (**Figures 8B, C**).

Moreover, the correlations between *AGTRAP* and T-cell exhaustion markers, such as PD-1 (PDCD1), GZMB, LAG-3, CTLA-4, and HAVCR2 (TIM-3) in HCC tissues were explored using the GEPIA database. The results demonstrated a positive relationship between *AGTRAP* expression and the five biomarkers of T-cell exhaustion (**Figure 8D**, all *p < 0.001*).

### Expression of AGTRAP Protein in HCC Tumor Tissues and Adjacent Tissues From Clinical Samples

**Figure 9:**
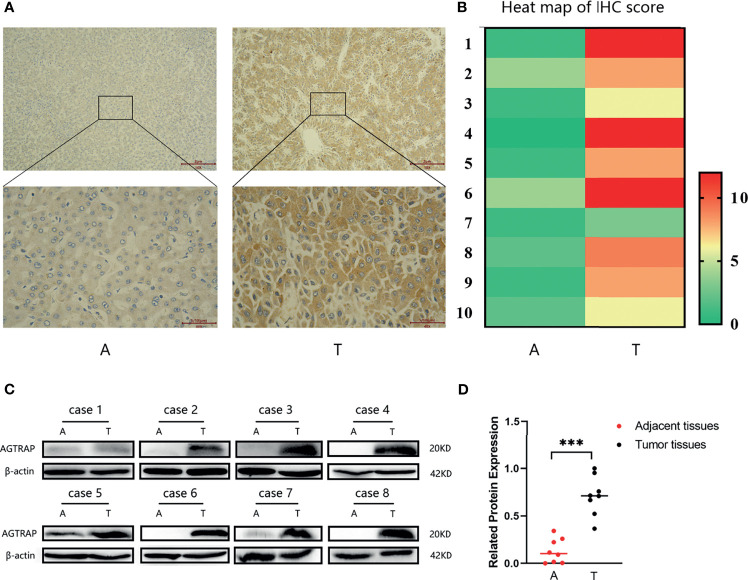
The protein expression of AGTRAP from HCC patients. **(A)** AGTRAP expression in HCC adjacent tissues and tumor tissues tested by IHC. **(B)** The heatmap of IHC score. **(C)** AGTRAP protein level was increased in HCC tumor tissues compared with paired adjacent tissues as measured by western blot. **(D)** A semi-quantitative analysis of expression differences between adjacent tissues and tumor tissues. (***p < 0.001, paired samples nonparametric test). A, adjacent tissues; T, tumor tissues.

We assessed the expression of AGTRAP protein in HCC tumor tissues (T) and paired adjacent tissues (A). The protein expression of AGTRAP was higher in tumor tissues than in adjacent tissues (**Figure 9A**) and was mainly localized in cell plasma. Moreover, the heat map showed that AGTRAP expression was higher in HCC tumor tissues than in adjacent tissues *(p < 0.001*) (**Figure 9B**). Western blot were also performed to validate the different expression of AGTRAP between adjacent tissues and tumor tissues in eight couple of paired samples, and the results (**Figures 9C, D**) showed that AGTRAP has a higher expression in tumor tissues than that in adjacent tissues (*p < 0.001*), which is consistent with our bioinformatic analysis and IHC results.

## Discussion

HCC continues to cause a serious public health burden worldwide. Most patients with HCC are diagnosed at an advanced stage when the tumor is unresectable (25). A need exists for the development of reliable biomarkers that would allow for early diagnosis of HCC and the accurate prognosis of long-term outcomes. In this study, we identified the diagnostic and prognostic value of *AGTRAP* in HCC, the mechanisms that might affect the development of HCC, and its association with immune infiltration and T-cell exhaustion.

A growing amount of research suggests that *AGTRAP* may play an important role in tumor progression (7, 26). Sanz-Pamplona et al. (7) performed an exome sequence of 42 cases of colon tumors and their paired mucosa and discovered that *AGTRAP* was overexpressed in colon cancer with poor prognosis. Zeng et al. (26) used weighted gene co-expression network analysis (WGCNA) and found that *AGTRAP* was overexpressed and was related to poor prognosis in tongue squamous cell carcinoma. Palanisamy et al. (27), through paired-end transcriptome sequencing, detected *AGTRAP-BRAF* gene fusion in gastric cancer. In concurrence with these studies, we found that mRNA and protein levels of *AGTRAP* were higher in HCC tissues compared to normal tissues, and our results were verified using clinical samples. In addition, our results showed that high expression of *AGTRAP* mRNA was associated with poor OS. Furthermore, the ROC curve indicated that *AGTRAP* is a sensitive diagnostic marker. Xiong et al. (28) identified *AGTRAP* as one of the 10 most notable prognosis genes in lower-grade glioma by WGCNA and survival analysis. Given these results, *AGTRAP* likely plays an important role in the occurrence and development of HCC.

Next, to better understand whether *AGTRAP* could affect the progression of HCC, we explored the association between *AGTRAP* and clinicopathological parameters in HCC. Consistent with our expectations, we found that patients with higher tumor grades had higher *AGTRAP* levels, accompanied by worse survival outcomes. However, this result was based on a small sample size of patients in stage T4 (n = 6). Additionally, our results demonstrated that the level of *AGTRAP* was significantly correlated with patient weight, especially BMI ≥ 30 kg/m^2^, suggesting that obese patients may have a higher risk of HCC. Indeed, obesity has already been associated with several types of cancer (29). Additionally, our results showed that patients with TP 53 mutation also displayed higher *AGTRAP* levels in HCC. Unexpectedly, there was no statistical difference in lymph node metastasis. This could be attributed to the small number of cases and therefore increased sample size would benefit future research. Although current knowledge suggests that *AGTRAP* plays an important role in tumor occurrence and development, more research is required to elucidate its effects on tumors.

Genetic alterations and dysregulated amplification are critical for the development of many tumors (30). There was an 8% genetic alteration rate of *AGTRAP* in HCC patients in the cBioPortal database, and its related genes were mainly involved in ubiquitin-like protein binding and the regulation of the actin cytoskeleton. Protein ubiquitination, which is a type of post-translational protein modification, has been shown to be widely involved in the invasion and metastasis of malignant tumors and that multiple ubiquitin-activating enzymes regulate the expression of key factors in various tumor metastasis-related pathways (31–33). However, changes in the cytoskeleton could affect the activity of NF-κB, which is known to affect the immune response and carcinogenic response (34, 35). Interestingly, our PPI results showed that *BRAF* and MAPK3 interact with *AGTRAP*, suggesting that *AGTRAP* may be related to the MAPK signaling pathway. This is also in accordance with *AGTRAP-BRAF* gene fusion detected in gastric cancer (8).

Immune infiltration is known to correlate with the prognosis of numerous cancers (36). Our KEGG analysis of genes co-expressed with *AGTRAP* in HCC tissues and adjacent tissues also showed that its co-expressed genes were mainly enriched in neutrophil degranulation and neutrophil activation. Our analyses, based on datasets from multiple recognized databases, found a positive correlation between *AGTRAP* expression and all immune cells as well as T-cell exhaustion in HCC patients based on TCGA data. Similarly, Xiong et al. (28) found that *AGTRAP* displayed potential as an immunotherapy target in brain lower-grade glioma because of its association with different immune infiltrating cells. Here, we found that *AGTRAP* might affect the tumor microenvironment by regulating immune cells, especially neutrophils and T cells, and may serve as an immunotherapy target in HCC.

Based on our bioinformatics analysis, it suggested that AGTRAP could be involved in the immune microenvironment of hepatocellular carcinoma, and related to MAPK signaling pathway. Many studies have implicated the MAPK pathway in both the development and progression of HCC and indicated that activation of the MAPK pathway correlates with a poor prognosis in human HCC (37, 38). In addition, Liao et al. (38)reported that CXCL8 secreted by HCC cells makes a tumorigenic inflammatory microenvironment to promote epithelial-mesenchymal transition and HCC invasion by MAPK pathway. Better understanding the immune subtypes of HCC could guide personalized immunotherapy and clinical management of HCC (39). And a recent study published in the journal of GUT has been identified that administration of p38 MAPK inhibitor could restrain monocytic myeloid-derived suppressor cell to suppress the proliferation of HCC, indicating that MAPK signaling pathway was closely related to immune microenvironment of HCC (40). Therefore, we speculate that AGTRAP may regulate immune microenvironment of hepatocellular carcinoma through MAPK signaling pathway. Besides, MAPK signaling pathway was known to affect the immune microenvironment of tumor by affecting macrophage polarization (41, 42). Yang et al. (43) illustrated that compound kushen injection activates macrophages form M2 to M1 *via* triggering TNFR1 and its downstream MAPK p38 signaling cascades, which induces HCC cells apoptosis and suppress HCC tumor growth and recurrence. Furtherly, we also design further mechanism research to verify whether AGTRAP could be involved in immune microenvironment of hepatocellular carcinoma by regulating MAPK signal pathway-induced macrophage polarization form M2 to M1.

In this study, we described the preliminary expression and possible mechanisms of action of *AGTRAP* in HCC. Further studies on the molecular mechanism of *AGTRAP* in HCC would be designed both *in vivo* and *in vitro*, as well as verified in a large number of clinical samples.

## Conclusion

The mRNA and protein levels of *AGTRAP* in HCC tissues were higher than those in normal liver tissues or adjacent tissues, and higher mRNA levels of *AGTRAP* were associated with higher histological grade and poor overall survival (OS) time in HCC patients. *AGTRAP* is involved in HCC, probably through the NF-κB and MAPK signaling pathways. Furthermore, *AGTRAP* positively correlated with immune infiltration and T-cell exhaustion. According to these results, *AGTRAP* could serve as a potential biomarker for prognostic, diagnostic, and even immunotherapy target for HCC.

## Data Availability Statement

The original contributions presented in the study are included in the article/**Supplementary Material**. Further inquiries can be directed to the corresponding authors.

## Ethics Statement

The studies involving human participants were reviewed and approved by The Ethics Committee of the First Affiliated Hospital of Chongqing Medical University. The patients/participants provided their written informed consent to participate in this study.

## Author Contributions

SL and NJ conceived and designed this study. SL and WZ collected and analyzed the relative data. XL collected the clinical samples. LZ and YG performed the IHC experiments. SL wrote the paper. QP and CD revised the manuscript. All authors contributed to the article and approved the submitted version.

## Funding

This project was supported by National Science Foundation of China (No. 97081252) and Science and Technology Research Foundation of Chongqing Municipal Education Commission (No. KJQN201900425).

## Conflict of Interest

The authors declare that the research was conducted in the absence of any commercial or financial relationships that could be construed as a potential conflict of interest.

## Publisher’s Note

All claims expressed in this article are solely those of the authors and do not necessarily represent those of their affiliated organizations, or those of the publisher, the editors and the reviewers. Any product that may be evaluated in this article, or claim that may be made by its manufacturer, is not guaranteed or endorsed by the publisher.

## Supplementary Material

Supplementary Figure 1 **(A)** The mRNA expression level of *AGTRAP* was significantly higher in HCC tissues than in normal tissues. **(B)** The expression level of *AGTRAP* was positively correlated with nodal metastasis in HCC patients.
