# Supplementary figures and images for: AGTRAP Is a Prognostic Biomarker Correlated With Immune Infiltration in Hepatocellular Carcinoma
Source: Front Oncol. 2021 Sep 14;11:713017. doi: 10.3389/fonc.2021.713017 (PMC8477650; doi:10.3389/fonc.2021.713017)

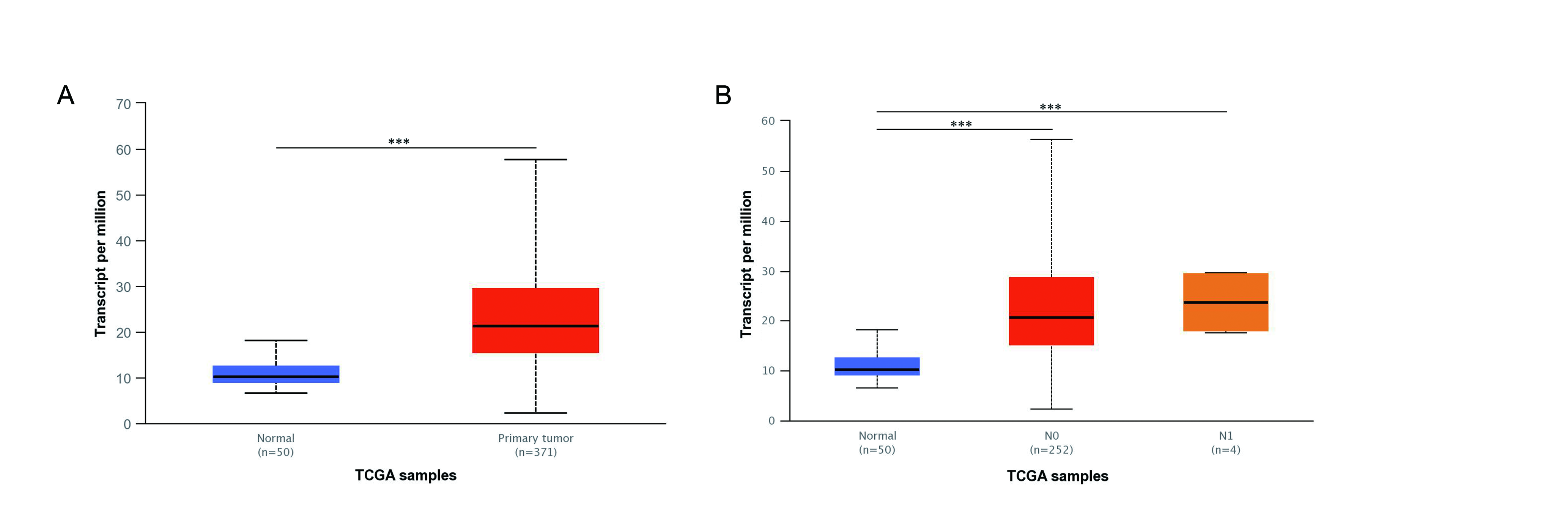

Supplement: Supplementary Figure 1 — (A) The mRNA expression level of AGTRAP was significantly higher in HCC tissues than in normal tissues. (B) The expression level of AGTRAP was positively correlated with nodal metastasis in HCC patients. [file Image_1.tif]
