# Supplementary material for: AGTRAP Is a Prognostic Biomarker Correlated With Immune Infiltration in Hepatocellular Carcinoma
Source: Front Oncol. 2021 Sep 14;11:713017. doi: 10.3389/fonc.2021.713017 (PMC8477650; doi:10.3389/fonc.2021.713017)
Supplement: Supplementary file 2 [file Table_1.doc]

**Table S1. Tools/databases used in this article.**

| **Tools/databases** | **link** | **application** |
| --- | --- | --- |
| **UCSC Xena** ^[11]^ | (https://xenabro wser.net/datapages/) | Provide raw data |
| **MERAV ^[12]^** | http://merav.wi.mit.edu | Analyze expressions of genes |
| **CCLE ^[13]^** | https://portals.broadinstitute.org/ccle | Analyze gene expression of cancerous cell lines in vitro |
| **HCCDB ^[14]^** | http://lifeome.net/database/hccdb | Analyze the expression of gene in different liver tissues and obtain the co-expression network |
| **HPA ^[15]^ (version 20.1)** | https://www.proteinatlas. org | Compare the expression of genes in different tissues and cells |
| **Kaplan-Meier plotter ^[16]^** | http://kmplot.com/analysis/ | Analyze the prognostic value of genes |
| **UALCAN ^[17]^** | ualcan.path.uab.edu | Analyze the relationships between gene expression and the clinicopathological parameters |
| **Linkedomics ^[18]^** | https://linkedomics.org/ | Plot data as heat maps, scatter plots, and volcano plots. |
| **cBioPortal ^[19]^**(version 3.7.1) | http://cbioportal.org | analyze the mutation of target gene and obtain its co-expression genes |
| **STRING** ^[20]^(version 11.5) | http://string-db.org | Demonstrate the functional networks of the gene |
| **TIMER** ^[21]^(version 1.0) | https://cistrome.shinyapps.io/timer/ | The association between gene expression and immune infiltration |
| **GEPIA** ^[22]^(version 1.0) | http://gepia.cancer-pku.cn/ | Expression analysis, correlation analysis, and survival analysis |
| **R** (version 3.6.3) | https://www.r-project.org/ | GO and KEGG enrichment analysis |
